# Impact of timing and format of patient decision aids for breast cancer patients on their involvement in and preparedness for decision making - the IMPACTT randomised controlled trial protocol

**DOI:** 10.1186/s12885-024-12086-z

**Published:** 2024-03-12

**Authors:** Bettina Mølri Knudsen, Stine Rauff Søndergaard, Dawn Stacey, Karina Dahl Steffensen

**Affiliations:** 1 Center for Shared Decision Making, Lillebaelt Hospital - University Hospital of Southern Denmark, Beriderbakken 4, 7100 Vejle, Denmark; 2 Department of Regional Health Research, Faculty of Health Sciences, University of Southern Denmark, Campusvej 55, 5230 Odense M, Denmark; 3 Department of Oncology, Lillebaelt Hospital – University Hospital of Southern Denmark, Vejle, Beriderbakken 4, 7100 Vejle, Denmark; 4 School of Nursing, University of Ottawa, 451 Smyth Rd, Ottawa, ON K1H 8M5 Canada; 5 Centre for Implementation Research, Ottawa Hospital Research Institute, 725 Parkdale Ave, Ottawa, ON K1Y 4E9 Canada

**Keywords:** RCT, Breast cancer, Shared decision making, Patient decision aid

## Abstract

**Background:**

After curative surgery for early-stage breast cancer, patients face a decision on whether to undergo surgery alone or to receive one or more adjuvant treatments, which may lower the risk of recurrence. Variations in survival outcomes are often marginal but there are differences in the side effects and other features of the options that patients may value differently. Hence, the patient’s values and preferences are critical in determining what option to choose. It is well-researched that the use of shared decision making and patient decision aids can support this choice in a discussion between patient and clinician. However, it is still to be investigated what impact the timing and format of the patient decision aid have on shared decision making outcomes. In this trial, we aim to investigate the impact of a digital pre-consult compared to a paper-based in-consult patient decision aid on patients’ involvement in shared decision making, decisional conflict and preparedness to make a decision.

**Methods:**

The study is a randomised controlled trial with 204 patients at two Danish oncology outpatient clinics. Eligible patients are newly diagnosed with early-stage breast cancer and offered adjuvant treatments after curative surgery to lower the risk of recurrence. Participants will be randomised to receive either an in-consult paper-based patient decision aid or a pre-consult digital patient decision aid. Data collection includes patient and clinician-reported outcomes as well as observer-reported shared decision making based on audio recordings of the consultation. The primary outcome is the extent to which patients are engaged in a shared decision making process reported by the patient. Secondary aims include the length of consultation, preparation for decision making, preferred role in shared decision making and decisional conflict.

**Discussion:**

This study is the first known randomised, controlled trial comparing a digital, pre-consult patient decision aid to an identical paper-based, in-consult patient decision aid. It will contribute evidence on the impact of patient decision aids in terms of investigating if pre-consult digital patient decisions aids compared to in-consult paper-based decision aids support the cancer patients in being better prepared for decision making.

**Trial registration:**

ClinicalTrials.gov (NCT05573022).

**Supplementary Information:**

The online version contains supplementary material available at 10.1186/s12885-024-12086-z.

## Introduction

Patient involvement in treatment decisions during consultation between patients and clinicians is an essential element for increasing the patient's quality of life and decision quality [1]. One approach to involve patients in decisions about their treatment is through shared decision making (SDM), which is a collaborative process between patient and clinician to find the best match between available treatment options and the patient’s informed preferences and values [2]. It is particularly in the field of cancer that SDM has gained momentum in recent years, as cancer treatment involves advanced treatment options, leaving the patient with difficult decisions, for example undergoing one or more adjuvant treatments if the risk of recurrence is minimal while considering the side effects and late effects caused by the adjuvant treatment.

While, survival rates and side effects are often decisive for the recommended treatment, less attention is paid to other long-term consequences and quality of life [3]. A recent study reported that cancer patients primarily require information on quality of life and the impact of side effects, while clinicians focus on the survivorship outcomes in their consultations. This emphasises the necessity for patients and clinicians to communicate about the possible treatment options including all benefits and harms [4]. Patients have different values or preferences for potential benefits and harms across options, making it preference-sensitive, but they may need guidance on the right choice for their circumstances.

SDM is an approach in which the clinician and patient work together to select treatments based on clinical evidence as well as the patient’s informed preferences [5]. SDM is particularly important in preference-sensitive decisions, where there is more than one clinically appropriate treatment option, each with benefits and harms, and in which the patient’s values and preferences are critical in determining the chosen intervention [6]. Due to the complexity and challenges within cancer care, this is an area requiring SDM. A cancer diagnosis is life-changing, and often cancer-related decisions made by the patient are affected by uncertainty, and emotions may negatively impact cognition [7]. Thus, many patients need support to make a high-quality decision based on informed clinical knowledge of available treatment options congruent with their preferences.

One way to support patients in their decision-making process and to inform them about the disease and its possible treatment options is by the use of patient decision aids (PtDAs), which are the most well-studied SDM interventions [8, 9]. PtDAs are tools designed to provide patients with evidence on harms and benefits of options, help them clarify what matters most to them, and empower them to make decisions. The use of PtDAs has been shown to create more realistic expectations of possible benefits and harms, improved knowledge, reduced decisional conflict, etc. [9]. However, there are barriers to patient participation in SDM [10, 11], and studies show that patient preparedness is an important factor; PtDAs are more helpful to patients actively considering their options versus those who have yet to start to think about their options or have already made a choice [12, 13].

PtDAs can be given to the patient before the consultation, thus facilitating preparation before discussing the decision with their clinician. In this case, tools can be interactive, digital solutions (apps, videos, websites etc.) or paper-based [9]. PtDAs primarily designed to be used independently by patients before their clinician visit – pre-consult tools – are widely evaluated in many different clinical settings. A 2024 Cochrane review of randomised trails reported widespread evaluation of PtDAs delivered pre-consult, and findings demonstrate that pre-consult tools increase preparedness to make decisions and reduce decisional conflict [9]. Authors argue that pre-consult tools provide patients with sufficient knowledge to participate constructively in decision-making [13, 14], and some patients prefer to assess the PtDA in the comfort of their own home, alone or with relatives [15].

PtDAs can also be designed primarily for clinicians and patients to use together within the consultation to structure the clinical counselling and facilitate SDM during the consultation [8, 14]. These in-consult tools help clinicians discuss treatment options and stimulate the integration of patient preferences into the decision-making process [14]. In-consult tools have shown to reduce decisional conflict, enhance the feeling of being involved in the decision-making process and establish a higher degree of shared or collaborative role when using in-consult PtDAs as opposed to a patient- or clinician-controlled role [9, 16, 17].

Although there is consistent evidence that pre-consult and in-consult PtDAs increase patient engagement, no randomised controlled studies compare them to determine whether one approach leads to better SDM outcomes [9, 18]. The 2024 Cochrane review established that there are knowledge gaps between the two about the timing of PtDA (before or during the consultation), patient-clinician communication (role and level of SDM) and format of PtDAs (digital or paper-based) [9]. In summary, little is known about the timing and format of a PtDA, and further research is critical given the increasing and widespread interest and investment in PtDAs [19].

Adding to this knowledge gap is the increasing use of health technologies, including digital PtDAs, which has accelerated over the past two decades and has various implications for patients. Digital health has improved quality, safety and efficiency in healthcare—but digital health also represents a transformational shift in how care and treatment are delivered [20]. Some patients do not cope well with this, and it is, therefore, essential that new digital solutions in health care, such as digital PtDAs, are evidence-based.

This study aims to explore the use of pre-consult digital PtDAs versus in-consult paper-based PtDAs to investigate if the format and timing of introduction of a PtDA for breast cancer patients in the decision making process have an impact on SDM, decisional conflict and preparedness to make decisions. The overall hypothesis is that the patients experience a higher degree of shared decision making when an in-consult PtDA is used.

## Methods and analysis

### Study design

The IMPACTT study is designed as a randomised, controlled trial with two arms in a 1:1 allocation ratio and a primary endpoint of patient-reported involvement in shared decision making. The study protocol is reported following the CONSORT guidelines [21] and registered at ClinicalTrials.gov (NCT05573022).

### Setting

Enrolment takes place at two Danish oncology outpatient clinics in the Region of Southern Denmark offering adjuvant treatment to breast cancer patients. The oncologists are the same at both sites, and they have received the same training in SDM and the use of the paper-based PtDA. During the consultations, an oncologist informs breast cancer patients on the adjuvant treatments options after undergoing curative surgery for early-stage breast cancer (e.g. chemotherapy or biologically targeted therapy).

### Eligibility criteria

Patients over 18 years of age with histologically verified early-stage breast cancer who had curative surgery are eligible to participate in the study. Patients are only eligible if they can read and understand Danish and have a smartphone or tablet to which it is possible to download an app (the digital PtDA).

Eligible patients are contacted by phone before the consultation in the outpatient oncology clinic to enable randomisation of patients willing to participate and to allow time for patients in the digital arm to access and use the digital PtDA before the consultation. Patients are therefore excluded if there are less than 24 h between the final consultation with the surgeons and the first consultation in the oncology outpatient clinic.

### Recruitment procedure

At study initiation, the oncologists will be asked to sign a written consent for audio recording the consultations when the adjuvant treatment options are discussed. The consent is only given once and includes all consultations with eligible patients throughout the study period.

**Fig. 1:**
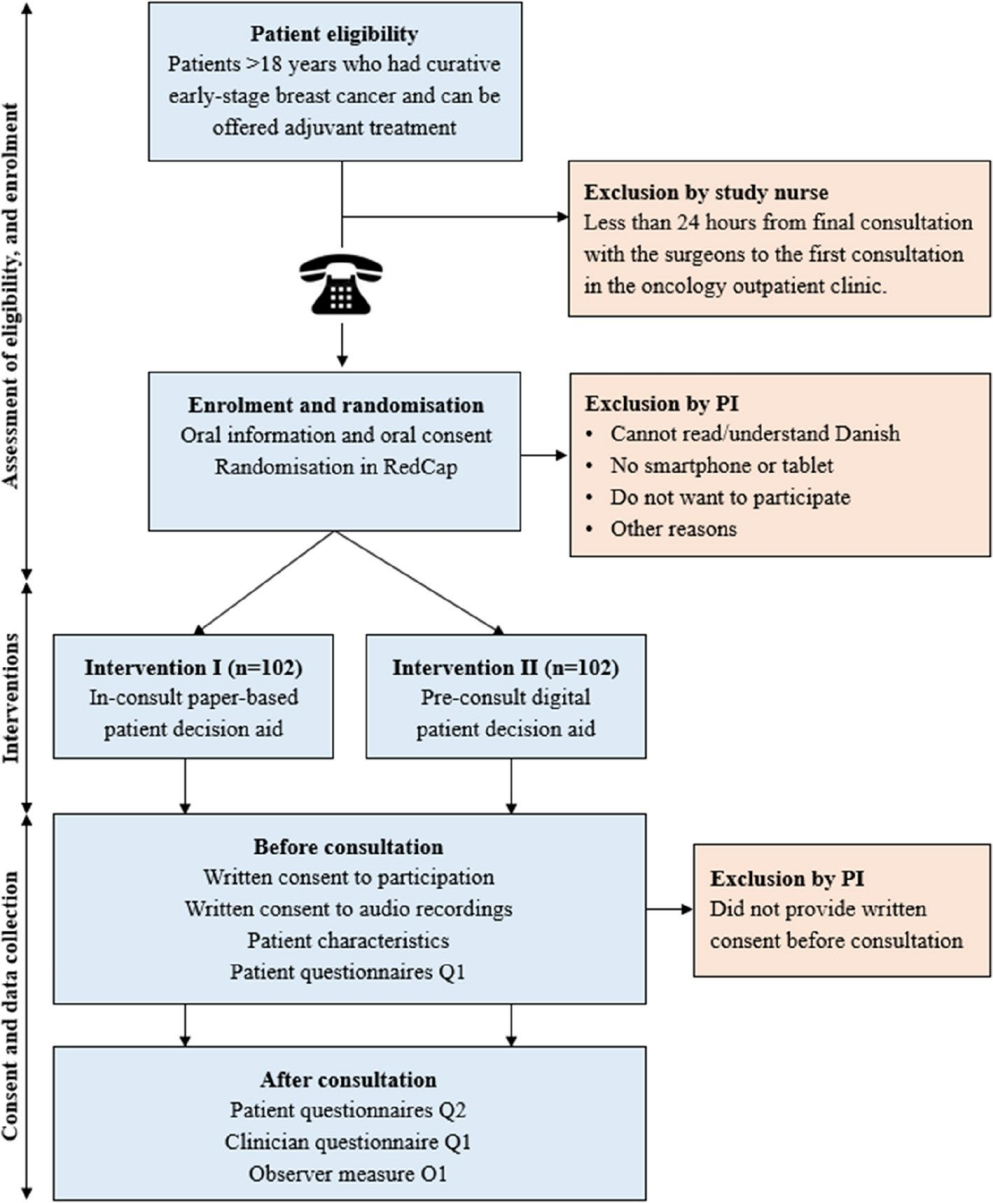
Recruitment procedure flow diagram with eligibility, interventions and data collection. Q: Questionnaire. O: Observer measure. PI: Principle investigator

Following standard procedures, patients with verified breast cancer are discussed at multidisciplinary team meetings (MDT) to identify the available management options. Eligible patients are then identified by a study nurse the day after the MDT based on the descriptive note from the MDT available in the electronic patient record. The note indicates: a) the relevant adjuvant treatments for the specific patient; b) when the patient is scheduled for the final consultation with the surgeon; and c) when the patient has their first consultation at the oncology outpatient clinic to discuss the available adjuvant treatment options (see Fig. 1).

The principal investigator telephones the eligible patients, identified by the study nurse, to obtain oral consent and randomise them. Randomisation will be performed as block randomisation with an equal allocation ratio (1:1) using a computer-generated table of block sizes of 4 or 6, created and supervised by a data manager at OPEN, Open Patient Data Explorative Network, Odense University Hospital, Region of Southern Denmark.

### Interventions

Intervention arm I will be introduced to a paper-based PtDA by the clinician in the consultation. Participants in intervention arm II will be invited to access the digital PtDA via an app before the consultation. The paper-based PtDA will not be used during consultation in intervention arm II. Both arms will be exposed to the same PtDA in terms of the lexical, visual and structural content, only the format (digital versus paper-based) is different.

The PtDA used in this study is based on the generic patient decision aid template developed and clinically tested by the Center for Shared Decision Making, Vejle Hospital, Denmark and based on the International Patient Decision Aid Standards (IPDAS) [22]. The PtDA is customised for use by patients with early-stage breast cancer who went through curative surgery. The PtDA specifically indicates the decision about having none, one or more adjuvant treatments depending on the patient's value assigned to survival rates, side effects, long-term consequences, and quality of life. The PtDA involves a five-step procedure to make explicit clarification of the patient’s values for adjuvant treatment and presents options A) chemotherapy, B) radiation, C) hormone therapy, D) antibody therapy, and E) no treatment including benefits and harms of each option, outcome probabilities, and personal stories describing experiences of others that are relevant to the decision at hand. The options offered to the patient depend on the patient’s age and tumor characteristics. The paper-based version has been available for use during consultations in the clinic since 2017 [23].

### Outcomes

The primary outcome is the extent to which patients are involved in a shared decision making process reported by the patient using the SDM Process_4 questionnaire [24]. This instrument has four items and demonstrated good acceptability with high response rates and very low missing data. In a national study of 10 different medical conditions, the SDM Process_4 did not show floor or ceiling effects. Calculations showed moderate to good reliability (ranging from 0.54 to 0.87), and retest reliability was moderate (0.64). The construct validity assessment indicated that higher scores correlated with better decision quality and that patients were less likely to think they had made the wrong decision [24].

Secondary outcomes, using well tested instruments, are the patient-perceived level of SDM [25], patient engagement [26], preparation for decision making scale [12], decisional conflict scale [7], and Control preferences Scale [27]. Additional secondary outcomes are consultation length, clinician-perceived level of SDM [28], and observer-perceived patient involvement in SDM [29, 30]. All questionnaires are validated and translated into Danish (see Table 1 for further details). Patients’ demographic characteristics will include age, gender, education, occupation, and living arrangements.

**Table 1:** Content of data collection outcome measures

Distribution	Measurement instrument	Description	Item no., range and scoring
Patient Q1	Decisional Conflict Scale [7]	Patient-reported perceived measure of four of five dimensions of decisional conflict (e.g., uncertainty, uninformed, unclear values, unsupported)	12 items 5 point Likert scale (0–4) Mean standardized score*25 (0–100)
	Control Preference Scale [27]	Patient-reported preference for the patient’s preferred role in shared decision making	1 item with five statements (pick one)
	Access to digital PtDA	Patients in intervention arm II are asked if they have accessed and used the digital PtDA	Yes/No – if no, to state the reason: • Cannot install the app • Cannot open the PtDA in the app • Problems with e-Boks • Other technical issues • Did not have the time • Do not want to see the options • Other cause / do not know
Patient Q2	Shared Decision Making Process 4 (SDM Process_4) [24]	Patient-reported engagement	4 items 4 point Likert scale (0–1, yes/no) Points are summed (0–4)
	Decisional Conflict Scale [7]	Patient-reported perceived measure of five dimensions of decision making	16 items 5 point Likert scale (0–4) Mean standardized score*25 (0–100)
	Shared Decision Making Questionnaire 9 (SDM-Q-9) [25]	Patient-reported experience measure of perceived level of involvement	9 items 6 point Likert scale (0–5) Mean score*20 (0–100)
	CollaboRATE [26]	Patient-reported measure of patient experienced involvement	3 items 10 point Likert scale (0–9) Mean score (0–9)
	Preparation for decision making scale [12]	Patient-reported measure of preparation for decision making	10 items 5 point Likert scale (1–5) Mean standardized score*25 (0–100)
	Access to paper-based PtDA	Patients in intervention arm II are asked if the clinician showed them the paper-based PtDA during consultation	Yes/No/Do not know
Clinician Q1	Shared Decision Making Questionnaire 9 Doctor (SDM-Q-doc) [28]	Clinician-reported measure of perceived level of involvement	9 items 6 point Likert scale (0–5) Mean standardized score*20 (0–100)
	Use of digital PtDA	Clinicians are asked if patients in intervention arm II took out their smartphone or tablet during consultation to use the digital PtDA	Yes/no
Observer O1	OPTION5 [28]	Observer-perceived patient involvement in shared decision making	5 items 5 point Likert scale (0–4) Mean standardized score*25 (0–100)`
Other	Length of consultation	Minutes based on the audio recordings	

### Sample size

According to the SDM Process_4 user guide, Sepucha and Fowler [24] suggest assumptions for the sample size calculations, including a common standard deviation of σ = 1 and a mean difference between intervention arm I and II at δ = 0.5*SD. Further, assuming a significance level of α = 0.05 and a statistical power of 1-β = 0.8 (80%), a total of 204 patients will be recruited in the study which included an extra 10% to compensate for missing data and dropouts.

For secondary outcomes, the observer-perceived patient involvement in SDM (OPTION5) will be used to analyze the audio recordings of the consultations between patient and clinician. A separate sample size calculation was conducted for this outcome; based on a previous RCT study with a mean OPTION5 score of 26.6 in the control arm [31], the calculation of sample size for the OPTION5 measure requires 35 patients in each arm to achieve 80% power (alpha at 0.05) to demonstrate a difference of at least 13.4 points (50% increase in observed level of SDM) [31]. Patients will be consecutively asked for permission to audio record the consultation until the required sample size is achieved; hence, no dropouts or missing data are expected. Therefore, a total of 70 audio recordings are required.

### Data collection

Study data will be collected and managed using the REDCap electronic data capture tool (copyright Vanderbilt University, version 12.0.33 [32]) hosted at OPEN (Open Patient Data Explorative Network, Odense University Hospital, Region of Southern Denmark). After oral consent, patients will be randomised via REDCap and are immediately sent an electronic questionnaire via e-Boks to be answered before the consultation (a total of 13 questions, Patient Q1 in Table 1). E-Boks is a secure national platform for digital, personal mail used by public authorities to citizens. All Danish citizens are required to use e-Boks but can be exempted from use if they fulfil specific requirements. If exempted from use, the patient will receive the questionnaire by mail. In the first questionnaire, enrolled patients give their written, electronic consent to participate and are asked to indicate if they consent to audio recording of the consultation. Patients in intervention arm II will be asked to checkmark if they have accessed and used the digital PtDA and, if not, to state the reason it was not used.

The day after the consultation with the oncologist discussing adjuvant treatment, enrolled patients will receive the second questionnaire from REDCap (a total of 42 questions, Patient Q2 in Table 1). Patients in intervention arm II will also be asked to checkmark if the clinician used the paper-based PtDA during consultation. The patient will receive a reminder to complete the questionnaire after three and six days.

Clinicians will be administered one 9-item questionnaire on paper which they are asked to fill in right after the consultation while they have the consultation fresh in mind (Clinician Q2, Table 1). Clinicians will also be asked if patients in intervention arm II unprovokedly took out their smartphone or tablet during the consultation to use the digital PtDA. Afterwards, answers will be registered manually in REDCap by the principal investigator.

Audio recordings of clinician and patient consultations (N = 70) are collected (Observer Q1, Table 1) for later analysis. The nurse participating in the consultation is responsible for initiating the recording.

### Timeline

Enrolment is estimated to be completed within a period of 20 months.

### Patient and public involvement

Two patient representatives, who are currently or have previously undergone cancer treatment themselves, will be involved as co-researchers in scoring the observer-perceived measure OPTION5 to investigate if patients identify elements of perceived significance in the consultations not noticed by the researchers. The rating team consisting of two researchers and two patient representatives will initially meet for training in scoring to ensure that they are calibrated in scoring the five themes of OPTION5. The 70 audio recordings will be divided between the two patient representatives, while the two researchers listen to and score all 70 audio recordings.

The process and the patients' influence on the analysis process will be documented in an impact log [33]. The log is completed with the patients, and the day's audio recordings are evaluated and debriefed. The entire process of involvement will be reported and documented using GRIPP2 [34]. The patient representatives will also be asked to complete the PPEET self-reported co-researcher questionnaire [35].

### Statistical methods

Data will be stored in a secure server in OPEN Analyse. Descriptive analyses will be performed to explore exchangeability between the two study arms. Data will be presented in a table describing the study population on key characteristics.

The main analyses will be performed as mixed effect models by incorporating the clinician as a random effect. If there is no significant variation, analyses will be performed using linear regression. The 95% confidence intervals will be estimated using bootstrapping. In case of a screwed distribution on key characteristics (non-exchangeability) between the study arms, multivariate analyses will be performed adjusting for potential confounding factors.

Primary and secondary outcomes will be analysed as sum scores and sub scores, and sub-analyses including stratification by sex and age groups. Continuous measures will be presented as mean with standard deviations (SD) or median with interquartile range (IQR) depending on if the findings are normally distributed?. Means will be compared using a 2-sided t-test, medians using a non-parametric K-sample test on the equality of medians. A 2-sided *p*-value of ≤ 0.05 will be used to determine significance.

The consultation length will be measured using the audio recordings and analysed descriptively.

All analyses will be performed as intention-to-treat analyses, and in case a patient did not access the app before consultation, per-protocol analyses will be performed as a sub-analysis. Missing data will be handled according to the scoring instructions of the instruments.

Audio recordings of clinician and patient consultations will be rated by a team consisting of the principal investigator, a second researcher, and two patient representatives. Before rating, an initial calibration between the raters will be performed to avoid inter-observer variation and to reach a scoring consensus. Any discrepancies will be resolved by agreement or a fifth rating. Audio recordings will be stored in a secure server at OPEN.

The team rating the audio recordings will be blinded such that raters will not know which arm the patients were randomised. Overall OPTION scores will be compared using the Wilcoxon-Mann–Whitney two-sample rank-sum test. Inter-rater reliability will be calculated using Interclass Correlation Coefficients (ICC). A reasonable threshold is above 0.6 [36].

## Perspectives

Due to national clinical guidelines on cancer treatment, patients are treated very similarly, and key decision time points are often the same. While the guidelines aim at offering all cancer patients the same treatment options, there is still a decision to be made for the individual patient based on their preferences. The increasing number of more advanced and individualised treatment options complicates the choice, and taking the patient’s situation and preferences into account when planning treatment is often necessary to achieve the best possible outcomes. This calls for more evidence-based tools to support patient involvement and to guide patients in making decisions about their own treatment with their clinician.

In this study, patient and clinician perceived level of involvement in SDM, as well as observed reported SDM, will produce a comprehensive assessment of the patient’s engagement in decision making. Findings should enable us to improve the format and timing of administering a PtDA to future patients. For the individual patient, the results may contribute to better decision quality with the potential of providing improved quality of life, as well as reducing overtreatment at the hospitals.

## Dissemination

The results of this study will be published in international peer-reviewed journals and presented at national and international conferences. Authorship is defined according to the recommendations for conduct, reporting, editing, and publication of scholarly work by the International Committee of Medical Journal Editors (ICMJE) [37].

The patient representatives involved as co-researchers will be offered authorship if their contribution agrees with the ICMJE recommendations or they will be acknowledged in the publication. As well, they will be offered active participation with poster or abstract presentations at national or international conferences with the principal investigator.

### Supplementary Information

**Supplementary Material 1.**

## Data Availability

The datasets used and/or analysed during the current study are available from the corresponding author on reasonable request.
