# Supplementary material for: Impact of timing and format of patient decision aids for breast cancer patients on their involvement in and preparedness for decision making - the IMPACTT randomised controlled trial protocol
Source: BMC Cancer. 2024 Mar 12;24:336. doi: 10.1186/s12885-024-12086-z (PMC10935979; doi:10.1186/s12885-024-12086-z)
Supplement: Supplementary file 1 — Supplementary Material 1. [file 12885_2024_12086_MOESM1_ESM.pdf]

Reporting checklist for randomised trial.

Based on the CONSORT guidelines.

		Reporting Item	Page Number
**Title and Abstract**			
Title	#1a	Identification as a randomized trial in the title.	1
Abstract	#1b	Structured summary of trial design, methods, results, and conclusions	2
**Introduction**			
Background and objectives	#2a	Scientific background and explanation of rationale	2
Background and objectives	#2b	Specific objectives or hypothesis	4
**Methods**			
Trial design	#3a	Description of trial design (such as parallel, factorial) including allocation ratio.	4
Trial design	#3b	Important changes to methods after trial commencement (such as eligibility criteria), with reasons	4
Participants	#4a	Eligibility criteria for participants	4
Participants	#4b	Settings and locations where the data were collected	4
Interventions	#5	The experimental and control interventions for each group with sufficient details to allow replication, including how and when they were actually administered	6
Outcomes	#6a	Completely defined prespecified primary and secondary outcome measures, including how and when they were assessed	7

Sample size	#7a	How sample size was determined.	9
Sample size	#7b	When applicable, explanation of any interim analyses and stopping guidelines	n/a
Randomization - Sequence generation	#8a	Method used to generate the random allocation sequence.	5
Randomization - Sequence generation	#8b	Type of randomization; details of any restriction (such as blocking and block size)	5
Randomization - Allocation concealment mechanism	#9	Mechanism used to implement the random allocation sequence (such as sequentially numbered containers), describing any steps taken to conceal the sequence until interventions were assigned	5
Randomization - Implementation	#10	Who generated the allocation sequence, who enrolled participants, and who assigned participants to interventions	5
Blinding	#11a	If done, who was blinded after assignment to interventions (for example, participants, care providers, those assessing outcomes) and how.	11
Blinding	#11b	If relevant, description of the similarity of interventions	n/a
Statistical methods	#12a	Statistical methods used to compare groups for primary and secondary outcomes	10
Statistical methods	#12b	Methods for additional analyses, such as subgroup analyses and adjusted analyses	10
Outcomes	#6b	Any changes to trial outcomes after the trial commenced, with reasons	n/a
**Results**			
Participant flow diagram (strongly recommended)	#13a	For each group, the numbers of participants who were randomly assigned, received intended treatment, and were analysed for the primary outcome	6
Participant flow	#13b	For each group, losses and exclusions after randomization, together with reason	6

Recruitment	#14a	Dates defining the periods of recruitment and follow-up	n/a
Recruitment	#14b	Why the trial ended or was stopped	n/a
Baseline data	#15	A table showing baseline demographic and clinical characteristics for each group	7
Numbers analysed	#16	For each group, number of participants (denominator) included in each analysis and whether the analysis was by original assigned groups	10
Outcomes and estimation	#17a	For each primary and secondary outcome, results for each group, and the estimated effect size and its precision (such as 95% confidence interval)	10
Outcomes and estimation	#17b	For binary outcomes, presentation of both absolute and relative effect sizes is recommended	10
Ancillary analyses	#18	Results of any other analyses performed, including subgroup analyses and adjusted analyses, distinguishing pre-specified from exploratory	10
Harms	#19	All important harms or unintended effects in each group (For specific guidance see CONSORT for harms)	11
**Discussion**			
Limitations	#20	Trial limitations, addressing sources of potential bias, imprecision, and, if relevant, multiplicity of analyses	n/a
Interpretation	#22	Interpretation consistent with results, balancing benefits and harms, and considering other relevant evidence	n/a
Registration	#23	Registration number and name of trial registry	2
Generalisability	#21	Generalisability (external validity, applicability) of the trial findings	n/a

Other information

Interpretation	#22	Interpretation consistent with results, balancing benefits and harms, and considering other relevant evidence	n/a
Registration	#23	Registration number and name of trial registry	2
Protocol	#24	Where the full trial protocol can be accessed, if available	n/a
Funding	#25	Sources of funding and other support (such as supply of drugs), role of funders	12

The CONSORT checklist is distributed under the terms of the Creative Commons Attribution License CC-BY. This checklist was completed on 23. August 2023 using https://www.goodreports.org/, a tool made by the EQUATOR Network in collaboration with Penelope.ai
